# Case Report: A Novel *EIF2B3* Pathogenic Variant in Central Nervous System Hypomyelination/Vanishing White Matter

**DOI:** 10.3389/fgene.2022.893057

**Published:** 2022-06-17

**Authors:** Parith Wongkittichote, Soe Soe Mar, Robert C. McKinstry, Hoanh Nguyen

**Affiliations:** ^1^ Division of Genetics and Genomic Medicine, Department of Pediatrics, Washington University School of Medicine, St Louis, MO, United States; ^2^ Division of Pediatric Neurology, Department of Neurology, Washington University School of Medicine, St Louis, MO, United States; ^3^ Mallinckrodt Institute of Radiology, Washington University School of Medicine, St Louis, MO, United States

**Keywords:** leukodystrophy, childhood ataxia with central nervous system hypomyelination/vanishing white matter, EIF2B3, ataxia, developmental regression

## Abstract

Leukodystrophies are a group of heterogeneous disorders affecting brain myelin. Among those, childhood ataxia with central nervous system hypomyelination/vanishing white matter (CACH/VWM) is one of the more common inherited leukodystrophies. Pathogenic variants in one of the genes encoding five subunits of EIF2B are associated with CACH/VWM. Herein, we presented a case of CACH/VWM who developed ataxia following a minor head injury. Brain magnetic resonance imaging showed extensive white matter signal abnormality. Diagnosis of CACH/VWM was confirmed by the presence of compound heterozygous variants in *EIF2B3*: the previously known pathogenic variant c c.260C>T (*p*.Ala87Val) and the novel variant c.673C>T (*p*.Arg225Trp). Based on the American College of Medical Genetics (ACMG) recommendations, we classified *p*.Arg225Trp as likely pathogenic. We report a novel variant in a patient with CACH/VWM and highlight the importance of genetic testing in patients with leukodystrophies.

## 1 Introduction

Inherited leukodystrophies (Bonkowsky et al., 2021) are a group of largely heterogeneous genetic disorders affecting brain myelin (Vanderver et al., 2015). Although individually rare, inherited leukodystrophies are collectively common with an estimated incidence of 1 in 4,700 live births (Soderholm et al., 2020). In pediatric population, inherited leukodystrophies cause significant morbidity and mortality, with a mortality rate of 34% and an average age at death of 8.2 years (Bonkowsky et al., 2010). Leukodystrophies can present at any age, ranging from infancy to adulthood (Bonkowsky et al., 2021). Disease progression rate and severity vary, even among affected individuals within the same family. The diagnosis of inherited leukodystrophies relies on clinical presentation and brain imaging. With the advancement in sequencing technology, genetic testing has become a part of diagnosis (Soderholm et al., 2020).

Among inherited leukodystrophies, childhood ataxia with central nervous system hypomyelination/vanishing white matter (CACH/VWM) is one of the more common leukodystrophies (van der Knaap et al., 1993). In the Netherlands, the estimated incidence is 1 in 80,000 live births (Hamilton et al., 2018). CACH/VWM is characterized by ataxia, loss of motor skills, and optic atrophy. The patient with CACH/VWM may present at various ages ranging from fetus to adulthood (Hamilton et al., 2018). Disease progression and age of death are partially correlated with the age of onset. Patients with an onset age of less than 4 years typically have more rapid progression and earlier death (Hamilton et al., 2018). The diagnosis of CACH/VWM is established by brain magnetic resonance imaging (MRI) and genetic testing. Pathogenic variants in *EIF2B1*, *EIF2B2*, *EIF2B3*, *EIF2B4*, and *EIF2B5*, encoding the five subunits of the eukaryotic translation initiation factor 2B (eIF2B), are found to be associated with CACH/VWM (Leegwater et al., 2001; van der Knaap et al., 2002).

Herein, we report a child with CACH/VWM who developed ataxia following minor head trauma. He was found to be a compound heterozygote for a known pathogenic variant and a novel variant that is classified as likely pathogenic.

## 2 Materials and Methods

### 2.1 Case Description

**FIGURE 1:**
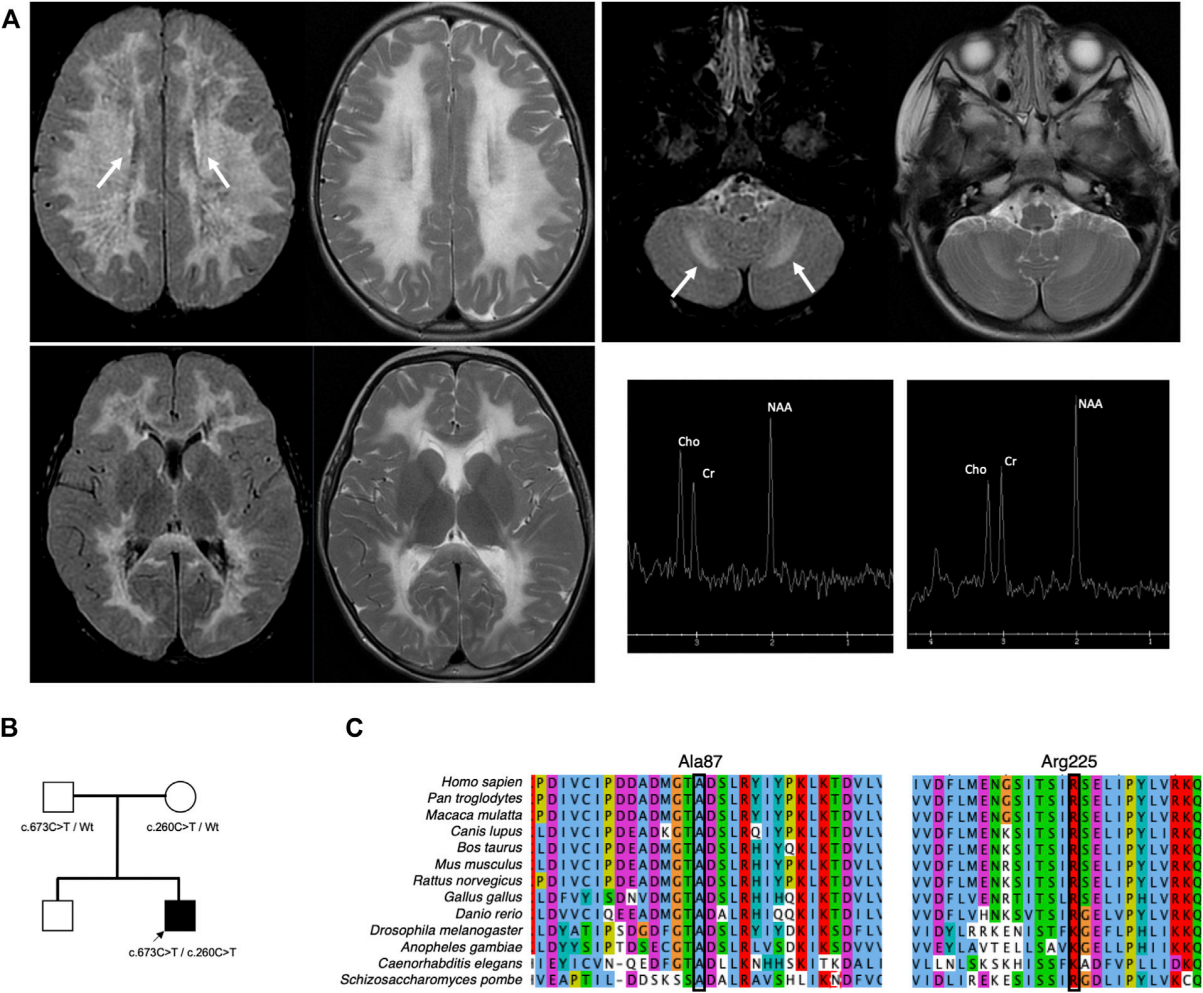
Brain imaging and pedigree of the proband. **(A)** T2-weighted MRI of the brain performed at 2 years and 11 months shows diffuse bilateral hyperintensity with a symmetrical distribution involving the supratentorial (left upper) and the cerebellar white matter (right upper) while sparing the basal ganglia (left lower). Elevation of the choline peak was detected on MR spectroscopy from the centrum semiovale but not from the basal ganglia (right lower). The proband inherited biallelic *EIF2B3* variants from both parents **(B)**. Both variants are highly conserved **(C)**.

Our proband is a male of Northern European ancestry who was born at term *via* vaginal delivery. Antenatal and neonatal courses were uncomplicated. He was meeting developmental milestones appropriately. At 2 years and 11 months, he had a minor accidental fall when he fell off the couch. The parents denied loss of consciousness; however, they noticed that he was unsteady. He remained unsteady for 3 h after the fall. His gait was improving over time; however, he remained unstable. He was referred to orthopedic surgery, and gait ataxia was confirmed. The child was referred to neurology for further evaluation. The initial examination was notable for positive Romberg signs with normal cranial nerve function, muscle tone and strength, cerebellar signs, and downgoing plantar reflex. Brain MRI revealed diffuse bilateral T2-weighted and FLAIR hyperintensity in a symmetrical distribution involving the supratentorial and the cerebellar white matter with sparing of the basal ganglia (Figure 1A). FLAIR hypointensity was detected around the ventricle, suggestive of tissue rarefaction. A mild elevation of the choline peak was detected on MR spectroscopy, while the lactate peak was absent. Diffusion-weighted imaging shows subtle diffusion restriction along the margins of the white matter signal abnormality. Enhancement was not detected following the administration of contrast. Cerebrospinal fluid analysis revealed normal glucose, protein, cell count, and differentiation and negative infectious studies.

Given the concern for leukodystrophy, he was referred to the genetics service. He was 3 years old at the first evaluation. His weight was at the 83rd percentile, and his height was at the 89th percentile. His occipitofrontal circumference (OFC) was at the 24th percentile. Neurological examination was notable for normal mental status, normal cognition, and normal speech content with mild dysarthria, mild tremor on the finger-to-nose test, mild hyperreflexia with normal functional motor strength, and wide-based gait, but he was able to ambulate without assistance for 10–12 steps. Biochemical profiles including lysosomal enzyme panel, urine ceramide trihexoside and sulfatide profiles, and leukocyte arylsulfatase A were normal.

### 2.2 Next-Generation Sequencing

Clinical targeted sequencing using the leukodystrophy panel was performed by Invitae (San Francisco, California). The variants were confirmed by Sanger sequencing. Bioinformatic analysis was performed by using combined annotation-dependent depletion (CADD) (Rentzsch et al., 2019). Variant classification was performed based on American College of Medical Genetics guidelines (Richards et al., 2015).

## 3 Results

Two variants were found in EIF2B3, designated as c.260C>T (*p*.Ala87Val) and c.673C>T (*p*.Arg225Trp). Parental testing confirmed *trans* configuration (Figure 1B). *In silico* analysis supported the deleteriousness of *p*.Ala87Val and *p*.Arg225Trp, with CADD scores of 26.4 and 32, respectively.

### 4 Follow-Up

Two months after the first evaluation, he developed worsening ataxia following 2 days of low-grade fever and upper respiratory infection symptoms. Interval changes in his examination included worsening hyperreflexia, extremity weakness, intension tremor, and very wide-based gait in addition to respiratory distress and wheezing on auscultation. He was admitted for dehydration and monitoring of respiratory status. He received a course of steroids for 5 days for presumed reactive airway disease. A follow-up examination demonstrated resolution of weakness and respiratory symptoms and improvement of his gait and reflexes. He was enrolled in the clinical trial shortly after his recovery from illness.

## 5 Discussion

Herein, we describe a child with CACH/VWM whose symptoms were triggered by minor head trauma. His MRI demonstrated diffuse T2 and FLAIR hyperintensity in cerebral white matter with secondary cavitation, which are typical findings of CACH/VWM. He has compound heterozygous variants, namely, *p*.Ala87Val and *p*.Arg225Trp. The variant *p*.Ala87Val is known to be pathogenic and is a founder variant among French–Canadians (Robinson et al., 2014). The residue Ala87 is highly conserved (Figure 1C). A novel variant, *p*.Arg225Trp, has been reported in one heterozygote in the population database (Karczewski et al., 2020). Although its pathogenicity was unclear, the genetic variant affecting this residue *p*.Arg225Gln has been reported to be pathogenic (van der Knaap et al., 2002). Overexpression of wild-type human EIF2B3 in *eif2b3* mutant zebrafish rescued morphological phenotypes; however, overexpression of human EIF2B3 harboring pathogenic variants, including *p*.Ala87Val and *p*.Arg225Gln, could not. The residue Arg225 is highly conserved (Figure 1C). *In silico* prediction supports that the change from Arg to Trp is likely to be disruptive (Rentzsch et al., 2019). Based on American College of Medical Genetics guidelines for variant classification, we classified *p*.Arg225Trp as likely pathogenic using the following criteria: PM2, PM3, PM5, PP3, and PP4 (Richards et al., 2015).

Our patient presented at the age of 3 years with ataxia following minor head trauma. The age of 2–4 years is the most common age of presentation in patients with CACH/VWM (Hamilton et al., 2018). The majority of the patients in this group have normal early milestones similar to our patient; only 9% have delayed early cognitive development (van der Knaap et al., 2022). The symptoms developed after the exposure to provocation factors, including fever, head trauma, and infection, in 72% of the patients in this group (van der Knaap et al., 2022). Approximately 90% of the patients have an exacerbated course after the exposure to provocation factors, in our patient upper respiratory tract infection (van der Knaap et al., 2022).

A recent study reported a case with CACH/VWM who received intravenous immunoglobulin and systemic corticosteroids with partial improvement in clinical and neuroimaging (Singh et al., 2017). Our patient received a short course of oral steroids primarily for respiratory symptoms with significant neurological improvement. Follow-up neuroimaging was not performed due to sedation risks. It is unclear whether the improvement was achieved by steroid therapy or as the natural progression of the disease. Corticosteroids have been used occasionally in patients with CACH/VWM (van der Knaap et al., 1993); however, the effect of immunomodulation in CACH/VWM has yet to be determined.

Five genes that encode eIF2B are associated with CACH/VWM in an autosomal recessive manner (Leegwater et al., 2001). Although certain *EIF2B5* variants are associated with milder or more severe diseases, the age of onset and survival are not significantly different among the patients with pathogenic variants in *EIF2B1*, *EIF2B2*, *EIF2B3*, *EIF2B4*, or *EIF2B5* (Hamilton et al., 2018). The patients who harbor the same pathogenic variants are likely to have similar disease courses, with the exception of milder phenotype, indicating a certain degree of genotype–phenotype correlation (Hamilton et al., 2018).

In eukaryotic cells, eIF2B acts as a guanine nucleotide exchange factor for eIF2. The formation of eIF2-GTP is required for translation initiation (Pavitt, 2005). eIF2B also plays a role in integrated stress response (ISR) (Marintchev and Ito, 2020). Stress-induced kinase phosphorylate eIF2, in turn, acts as a competitive inhibitor to eIF2B. Inhibition of eIF2B activates ISR which promotes both proapoptotic and pro-survival pathways (Marintchev and Ito, 2020). Dysregulated ISR plays a key role in CACH/VWM. Clinical trials for CACH/VWM are developed to target proteins in these pathways (van der Knaap et al., 2022).

In the past, the diagnosis of leukodystrophies relies mostly on the clinical course and brain imaging. Brain MRI is a crucial diagnostic step and helps determine the subgroup of leukodystrophies; however, the characteristics of brain MRI may not be able to distinguish specific leukodystrophies (Bonkowsky et al., 2021). The recent rapid development of next-generation sequencing facilitates the diagnosis of rare inherited leukodystrophies. A recent study demonstrates that whole-exome sequencing in patients with persistently unresolved white matter abnormalities yielded a diagnostic rate of 42% (Vanderver et al., 2016). We expect that the availability of clinical genome sequencing will increase the yield of genetic diagnoses in patients with leukodystrophies.

In conclusion, we report a patient with leukodystrophy. CACH/VWM was diagnosed based on targeted sequencing, elucidating the role of genetic diagnosis in patients with leukodystrophies.

## Data Availability

The original contributions presented in the study are included in the article/Supplementary Material; further inquiries can be directed to the corresponding author.
